# Synthesis, Characterization and Properties of Antibacterial Polyurethanes †

**DOI:** 10.3390/polym14010213

**Published:** 2022-01-05

**Authors:** Jihua Duan, Guichang Jiang

**Affiliations:** 1 College of Light Industry Science and Engineering, Tianjin University of Science and Technology, Tianjin 300222, China; djh@163.com; 2 Department of Chemistry, Tsinghua University, Beijing 100084, China

**Keywords:** polyurethanes, morphology, electron microscopy, self-assembly

## Abstract

Novel physically crosslinked polyurethane (PUII), based on isophorone diisocyanates, was prepared by a conventional two-step method. The chemical structures of the PUII were characterized by fourier transform infrared (FTIR), proton nuclear magnetic resonance (^1^H NMR), gel permeation chromatography (GPC), scanning electron microscopy (SEM) and DSC. The PUII hydrogels were subjected to solvent-induced self-assembly in THF + water to construct a variety of morphologies. The self-assembly morphology of the PUII was observed by scanning electron microscopy (SEM). The PUII films with different amounts (0.2%, 0.4%, 0.6%, 0.8%, 1.0%) of 1,3,5-Tris(2-hydroxyethyl)hexahydro-1,3,5-triazine (TNO) were challenged with *Escherichia coli*, *Staphylococcus aureus*, *Bacillus subtilis* and *Gray mold*. The results showed that when a small amount of antibacterial agent were added, the antibacterial effect of films on Botrytis cinerea was more obvious. The mechanical evaluation shows that the antimicrobial polyurethane films exhibit good mechanical properties.

## 1. Introduction

Polyurethanes (PUs) are one of the crucial classes of polymers with wide applications and properties, because their properties can easily be tailored by varying the components from which they are constructed [1,2,3,4,5,6,7]. Polyurethanes offer excellent versatility in terms of their mechanical properties and are widely applied in many fields [8,9,10,11,12,13,14,15,16,17,18,19]. In general, the synthesis of polyurethanes is carried out in two stages. The first stage is the synthesis of a prepolymer containing free isocyanate groups and in the second stage there are reactions of chain extension or crosslinking of the polymer chains. PUs are composed of hard segments and soft segments. In the hard segments, the polar groups of urethane form hydrogen bonds. The hard domains act as physically cross-linked sites and thus reinforce the PUs’ structure. However, the morphology and mechanical properties depend heavily on the kinds of soft segments [2]. Self-assembled polyurethanes (PUs) are a special class of biodegradable and biocompatible materials that are currently used in tissue engineering and drug delivery applications [18]. Polyurethanes exhibit many desirable characteristics for their diverse applications in coatings, adhesives, sealants, elastomers and plastics [20].

Traditionally, PUs are compounds of diisocyanate monomers such as 4,4-diphenylmethane diisocyanate (MDI) and polyatomic alcohol such as PTMG [21,22]. The polyurethanes obtained with these diisocyanates are easily oxidized and this causes the color change of the products. Under ultraviolet light, a urethane bond is easily damaged and ethyleniminoquinone is generated, so we always find that the color of PUs made by MDI turns yellow [23]. Compare with MDI, isophorone diisocyanates (IPDI) has excellent light stability and chemical resistance [24]. It is generally used in the manufacture of high-grade polyurethane resin. Traditionally, PUs are industrially produced by reacting petroleum-based polyols with isocyanates. The traditional synthesized polyurethane has high elasticity and strong bonding characteristics [25]. It can be used for the production of polyurethane adhesives. This kind of adhesive can be used for bonding metal, plastic, paper, wood and other materials. In the packaging field, the polyurethane adhesives are mainly used for bonding two or more layers of plastic film, especially dry composite film. The composite plastic film was mainly used in packaging pharmaceuticals, food and other goods. In addition, the polyurethane adhesives can also be used for the composition of paper and plastic [26]. In the biomedical packaging area, polyurethane has been used to develop polyurethane adhesives; however, no publication indicates its use in packaging products with polyurethane [27]. Hence, it is very important to investigate the possibility of the application of antimicrobial polyurethane film to package biomedical product. However, pure polyurethane has little antibacterial activity and it is easy to breed bacteria when the external environment is suitable. It is necessary to improve the antibacterial activity of polyurethane by incorporating antibacterial agents. Abbas Mohammadi [28] introduced Silver into a Schiff base ligand extended polyurethane to obtain a highly stable antibacterial dispersion. Kai Liu [29] modified polyurethane with lysozyme. Shazia Muzaffar [30] modified polyurethane with low molecular weight chitosan. In addition, TNO is also a good antibacterial agent. In this work, TNO was incorporated into polyurethane. In a previous publication, we used a macroiniferter-controlled radical polymerization method to successfully synthesize novel fluorinated polyurethanes and showed their interesting biological and mechanical properties that merit potential applications as biomaterials for tissue engineering [31].

Copolymer self-assembly behavior has been a hot research topic in recent years. By the structure or properties of different chain segments through chemical bond connected and block copolymers, microphase separation phenomenon will happen under certain conditions, and the formation of the various dimensions of nanometer or submicron structure will form. Self-assembly process and the formation of structure can be controlled by the chain segment of the chemical structure [32,33,34], chain length, the regulation of the connecting way [35,36] and the category and function of the external force field [37,38]. Self-assembly in the classic sense can be defined as the spontaneous and reversible organization of molecular units into ordered structures by non-covalent interactions. Self-assembled polyurethanes (PUs) are currently used in tissue engineering and drug delivery applications [18].

In this study, a novel physically crosslinked polyurethane (PUII) was synthesized by utilizing isophorone diisocyanates. In order to suppress interference of other substance, we synthesize PUII without dissolvent. The final products are characterized by FTIR, ^1^H NMR, GPC and SEM. Finally, the assembly behavior of PUII was investigated by SEM. The biocidal activity was examined by using the inhibition zone method.

## 2. Experimental Methods

### 2.1. Materials

IPDI (Aladdin, Shanghai, China) was used without further purification. Polytetramethylene oxide (PTMG, Mw = 2000) (Aladdin, Shanghai, China) was dried at 90 °C in vacuum for 48 h. 1,4-Dihydroxybutane (BDO) (Aladdin, Shanghai, China) and tetrahydrofuran (THF) (Aladdin, Shanghai, China) were used as received. 1,3,5-Tris(2-hydroxyethyl)hexahydro-1,3,5-triazine (TNO) (Aladdin, Shanghai, China) was dewatered before use. All other reagents (Aladdin, Shanghai, China) and solvents (Aladdin, Shanghai, China) were used as received without further purification.

### 2.2. Preparation of PUII

**Scheme 1:**
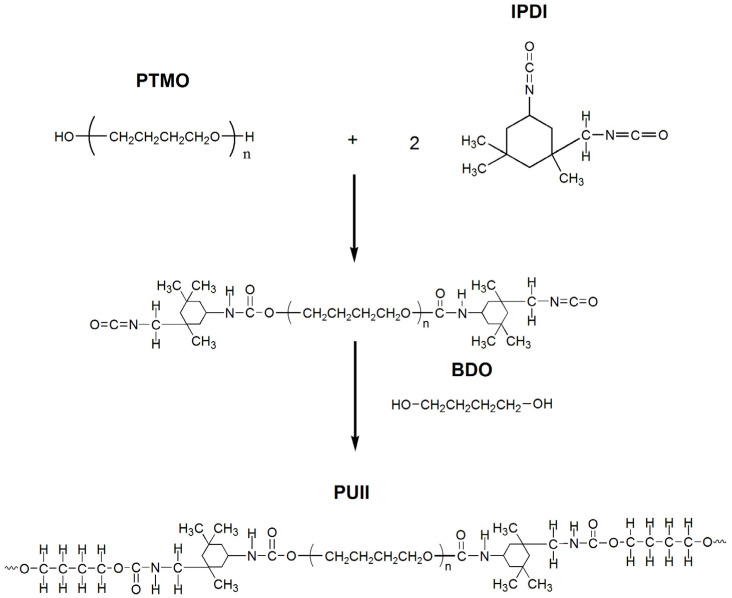
Synthesis route of polyurethane (PUII).

**Figure 1:**
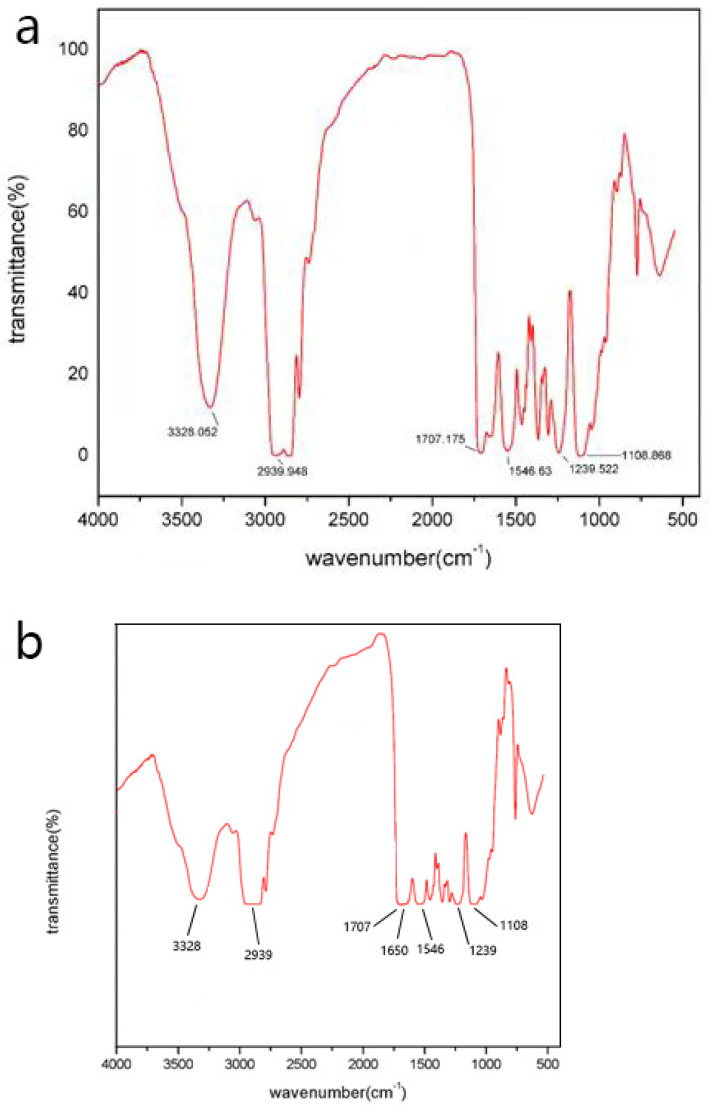
FTIR spectrum of polyurethane (PUII) (**a**), and polyurethane (PUII) with antibacterial agent (**b**).

**Figure 2:**
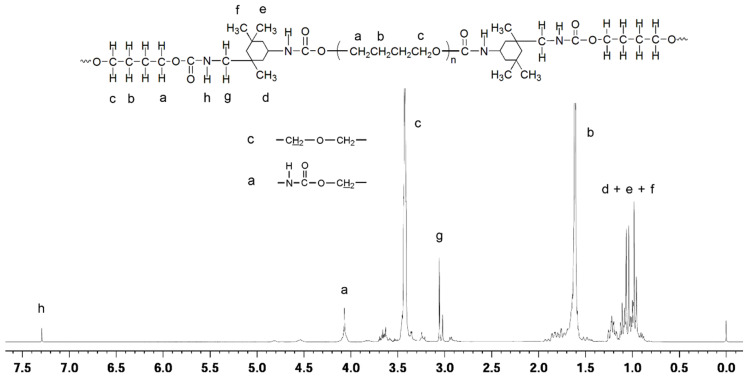
^1^H NMR spectrum of polyurethane (PUII).

PUII was prepared by two-step addition polymerization. A 250 mL glass reactor equipped with a heating element, a mechanical stirrer, a charging and sampling port, and a nitrogen inlet and outlet was charged with polytetramethylene oxide 2000 (15 g, 0.0075 mol) and isophorone diisocyanates (4 g, 0.018 mol) in a 1:2.4 mol ratio. The mixture was heated to 60–70 °C and allowed to react for 3 h. After 3 h, a small amount of BDO as chain extender was added and allowed to react for 60 min. Samples were frequently taken for FTIR analyses and reaction was allowed to proceed until the peak relating to the isocyanate (2265 cm^−1^) disappeared from the FTIR spectrum. The PUII was precipitated by pouring it into a water–methanol mixture (1:3 *v*/*v*) and dried at 30 °C in a vacuum oven (Scheme 1). Molecular weight: 7.9 × 10^4^ g/mol (*M*n). IR (Figure 1a): 3328 cm^−1^ (–NH), 2939 cm^−1^ (–CH), 1707 cm^−1^ (C=O), 1546 cm^−1^ (–CN), 1239 cm^−1^ (C–O–C, PUII), 1108 cm^−1^ (C–O–C, PTMG). ^1^H NMR (The spectrum was taken in CDCl_3_, Figure 2): δ = 0.96–0.98 ppm (d, e, f, CH_3_, IPDI), 1.62–1.63 ppm (b, CH_2_, PTMG), 3.06 ppm (g, CH_2_, IPDI), 3.40–3.41 ppm (c, CH_2_O, PTMG), 4.06–4.07 ppm (a, OCH_2_, PTMG), 7.29 ppm (h, NH, PUII).

### 2.3. Preparation of PUII with Antibacterial Agent

PUII with antibacterial agent was prepared by a one-step addition. Different amounts (0.2%, 0.4%, 0.6%, 0.8%, 1.0%) of 1,3,5-Tris(2-hydroxyethyl) -hexahydro-1,3,5-triazine (TNO) was added as antibacterial agent to the PUII and allowed to stir for 60 min. A solvent–casting method was used to prepare films of PUII with antibacterial agent. Specifically, about 10 mL dispersion was poured into a Teflon mold and dried at room temperature for one week. Finally, the films were dried at 45 °C for one day and then sealed for storage. IR (Figure 1b): 3328 cm^−1^ (–NH), 2939 cm^−1^ (–CH), 1707 cm^−1^ (C=O), 1650 (C–N–C, TNO), 1546 cm^−1^ (–CN), 1239 cm^−1^ (C–O–C, PUII with antibacterial agent), 1108 cm^−1^ (C–O–C, PTMG).

### 2.4. Characterization

IR spectra were recorded with a Bruker-Vector 22 FTIR spectrophotometer (Billerica, MA, USA) using KBr disks. Nuclear magnetic resonance (^1^H NMR) spectra were recorded in CDCl_3_ on a Bruker AVANCE III 400 MHz apparatus. The molecular weights and their distributions of the polymers were determined by gel permeation chromatography (GPC) utilizing a Waters model 515 pump and a model 2410 differential refractometer with three styragel columns HT2, HT3 and HT4 connected in a serial fashion. THF was used as the eluent at a flow rate of 1.0 mL/min. Polystyrene standards with dispersity of 1.08–1.12 obtained from Waters were employed to calibrate the instrument. Scanning electron microscopy (SEM) measurements were carried out using a JEOL JSM-5600 LV SEM (Tokyo, Japan). The samples were fabricated by dropping one blob of the mixed solution of PUII, THF and water onto a glass patch, and then evaporated for 48 h under ambient temperature. Finally, the samples were coated with gold and observed by SEM. The DSC studies of the samples were carried out using a DSC8000 (TA instrument, New Castle, DE, USA). The samples were dried in a vacuum oven at 50 °C for 28 h before use. The samples weighing between 5 and 15 mg were sealed in a DSC pan and quenched to −70 °C. The samples were then left to equilibrate for 15 min and heated to 200 °C at a rate of 5 °C/min. The mechanical properties of the samples were measured on a universal testing machine (GT-TS-2000, Taipei, Taiwan) according to GB/T 1040-2006 with a tensile speed of 200 mm/min at 23 ± 2 °C to obtain the tensile strength and the breaking elongation [39]. The samples were casted from DMF solutions and shaped to strip products. The thickness and width of the specimens were 3.0 and 10 mm, respectively. The length of the sample between the two pneumatic grips of Testing Machine was 50 mm. Five measurements were conducted for each sample, and the results were averaged to obtain a mean value.

### 2.5. Barrier Properties Experiment

The transmittance/haze of the samples was measured on a transmittance/haze meter (WGT-S, Suzhou, China) according to GB2410-80 at 23 ± 2 °C to obtain the transmittance value and the haze values. The samples were shaped to square products. The length and width of the specimens were 50 mm. Three measurements were conducted for each sample, and the results were averaged to obtain a mean value. The oxygen permeability of the samples [39] was measured on a permeability tester (GDP-C, Berlin, Germany) according to GB/T1038-2000 at 23 °C to obtain the oxygen permeability coefficient. The samples were shaped to square products. The length and width of the specimens were 120 mm. Four measurements were conducted for each sample, and the results were averaged to obtain a mean value. The water vapor permeability of the samples was measured on a Breathable Cup (0.003 m^2^, Chengde, China) according to GB/T1037-88 at 23 ± 2 °C to obtain the permeability coefficient. The samples were shaped to 38 mm radius circular products. Three measurements were conducted for each sample, and the results were averaged to obtain a mean value.

## 3. Results and Discussion

### 3.1. Synthesis of PUII

Novel physically crosslinked polyurethane (PUII) based on isophorone diisocyanates was prepared by a conventional two-step method. PUII was prepared according to the procedure shown in Scheme 1. The chemical structure of the final polymer (PUII) was characterized with FTIR in KBr matrices. The FTIR spectra of PUII are shown in Figure 1, which demonstrate the presence of the expected functional groups. As shown in Figure 1, peaks at 3328 cm^−1^ (–NH), 2939 cm^−1^ (–CH), 1707 cm^−1^ (C=O), 1546 cm^−1^ (–CN), 1239 cm^−1^ (C–O–C, PUII) and 1108 cm^−1^ (C–O–C, PTMG) are observed, which demonstrates that the synthesis is successful.

^1^H NMR spectrum of the PUII is shown in Figure 2. In the ^1^H NMR spectrum of PUII (Figure 2), a peak corresponding to the OCH_2_ (a) of the PTMG, was observed at 4.06–4.07 ppm. A peak related to the CH_2_ (b) of PTMG appeared between 1.62 and 1.63 ppm. Other peaks are listed as follows: 3.40–3.41 ppm (c, CH_2_O, PTMG), 0.96–0.98 ppm (d, e, f, CH_3_, IPDI), 3.06 ppm (g, CH_2_, IPDI) and 7.29 ppm (h, NH, PUII). This confirms the successful incorporation of IPDI to the polyurethane backbone.

GPC results indicate that the materials do not contain oligomers, and the PUII has molecular weight distributions (weight–average molecular weight of 72,000–79,000 and polydispersity of 1–2).

### 3.2. Self-Assembly Behavior of PUII

**Figure 3:**
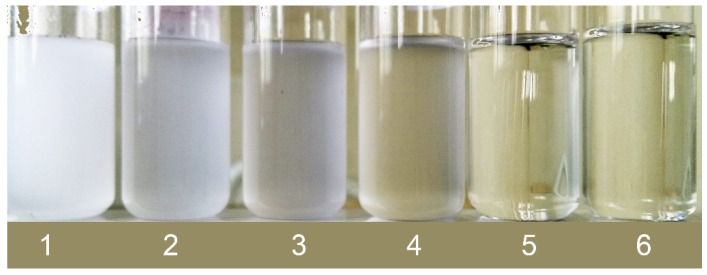
Samples of PUII self-assembly behavior (THF:water = 90:10/80:20/70:30/60:40/50:50/40:60, *v*/*v*, corresponding serial number: 1–6).

The self-assembly behavior of PUII was investigated by dissolving it in a mixed solution of THF and water. Six samples (THF:water = 90:10/80:20/70:30/60:40/50:50/40:60, *v*/*v*, corresponding serial number: 1–6) were prepared for SEM measurement. As shown in Figure 3, the samples became more pellucid due to the quantity of water decreasing. The samples were fabricated by dropping one blob of the mixed solution of PUII, THF and water onto a glass patch, and then evaporated for 48 h under ambient temperature. Finally, the samples were coated with gold and observed by SEM.

**Figure 4:**
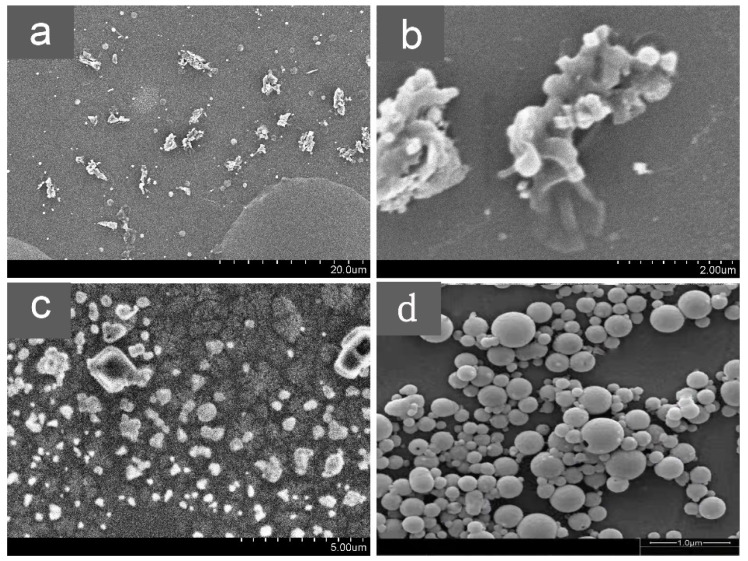
SEM images of PUII self-assembly behavior (The samples of number 6 and 1 (THF:water = 40:60/90:10) are correspondingly shown in (**a**,**c**). (**b**,**d**), respectively, reveal particulars of number 6 and 1).

As shown in Figure 4a–d, the SEM images of samples demonstrate that the polymer creates irregular globular morphologies. The average particle diameter was calculated by SEM images and the sizes range from 1 μm to 7 μm. The samples of number 6 and 1 (THF:water = 40:60/90:10) are correspondingly shown in Figure 4a,c. Figure 4b,d, respectively, reveal particulars of number 6 and 1. The space between irregular globular morphologies is gradually diminished when the quantity of water increases. So, water quantity plays an important role in the self-assembly behavior. In this study, number 5 (THF:water = 50:50) is a critical value. When the ratio of THF/water moves over this point, the aggregation becomes uncontrollable. We have created micron materials with good reproducibility by a solvent-induced assembly process, as confirmed by microscopic techniques of SEM. This is the first time that such a variety of morphologies of PU without dissolvents based on isophorone diisocyanates is found in the literature.

### 3.3. Barrier Properties of PUII

Different amounts of antimicrobial agents result in different outcomes. The antimicrobial PUII films with different amounts (0.2%, 0.4%, 0.6%, 0.8%, 1.0%) of 1,3,5-Tris(2-hydroxye-thyl)hexahydro-1,3,5-triazine (TNO) were measured for the haze and transmittance. The results are presented in Table 1. With an increase in the amounts of antimicrobial agents, transmittance of the films reduced and the haze of the films increased. The transparency of the antibacterial PUII films was lower than that of the PUII films.

**Figure 5:**
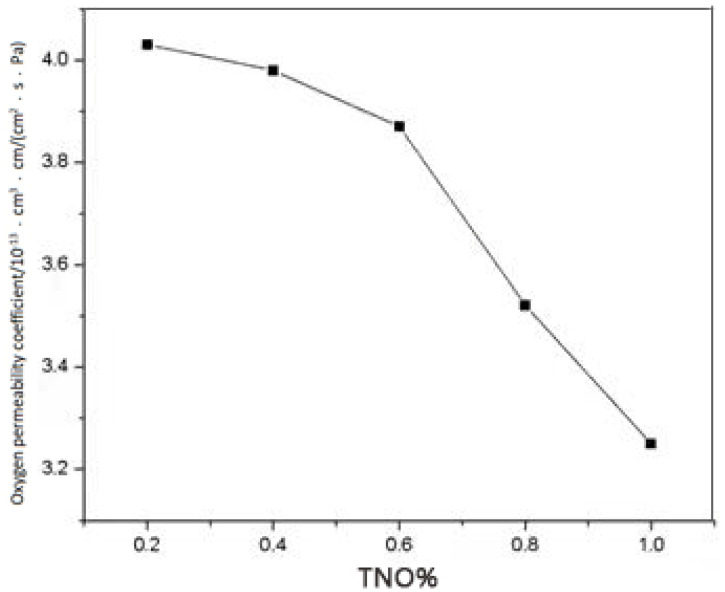
The oxygen permeability coefficient of the antibacterial PUII films.

**Figure 6:**
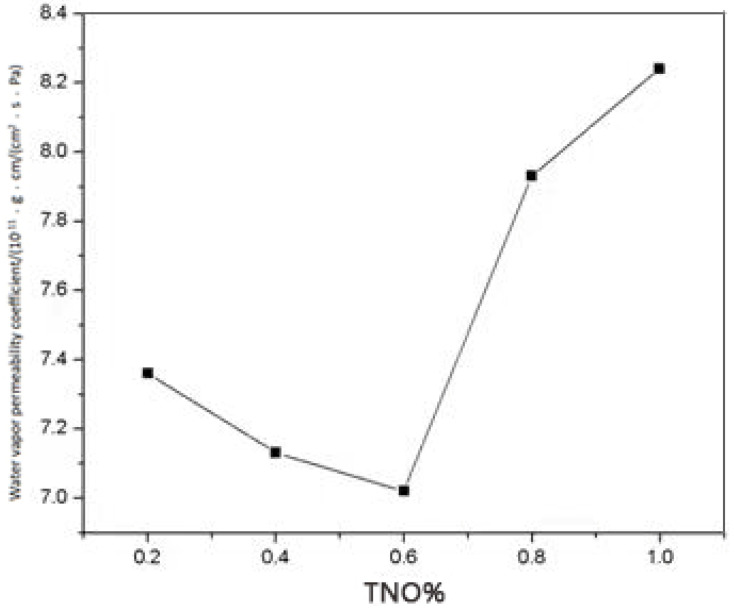
The water vapor permeability coefficient of the antibacterial PUII films.

Oxygen permeability testing results of the antibacterial PUII films presented in Figure 5. Oxygen permeability coefficients of the antibacterial polyurethane films was higher, and it decreased with an increase in the amounts of antimicrobial agents. The reason is that the antimicrobial agents mixed into the polyurethane physically. The antimicrobial agents attached to the surface of the film and formed a protective film. The variation of oxygen permeability stabilized slightly when the amount of antimicrobial agents arrived 1% gradually. The water vapor permeability testing results of the PUII films with different amounts of antimicrobial agents are shown in Figure 6. The water vapor permeability coefficient of the antibacterial PUII films decreased first, and it increased with an increase in the amount of antimicrobial agents (0.8%, 1.0%). The reason is that antimicrobial agents mixed into the polyurethane physically. The antimicrobial agents attached to the surface of the film and formed a protective film.

### 3.4. Mechanical Properties and Thermal Analysis of PUII

The mechanical properties of the PUII films and the antimicrobial PUII films were measured on a universal testing machine (GT-TS-2000, Taiwan) according to GB/T 1040-2006 with a tensile speed of 200 mm/min at 23 ± 2 °C to obtain the tensile strength and the breaking elongation. The tensile strength and the breaking elongation of the PUII films and the anti-microbial PUII films with different amounts (0.2%, 0.4%, 0.6%, 0.8%, 1.0%) of 1,3,5-Tris(2-hydroxyethyl)hexahydro-1,3,5-triazine (TNO) are shown in Table 2. The tensile strength of the PUII films increased as the amounts of the antibacterial agent increased. The tensile strength of conventional PE films is about 20–40 MPa, and the breaking elongation of conventional PE films is about 300–500%. The tensile strength and the breaking elongation of the PUII films and the antimicrobial PUII films are higher than that of the PE film, which suggests that the PUII films and the antimicrobial PUII films showed better mechanical properties in comparison with PE films. The PUII films and the antimicrobial PUII films possess higher tensile strength and breaking elongation compared to PVC films. Thermal analyses of the PUII films and antimicrobial PUII films were measured by DSC. The DSC studies of the PUII films and antimicrobial PUII films were carried out using a DSC8000 (TA instrument, New Castle, DE, USA). The PUII films and the antimicrobial PUII films were dried in a vacuum oven at 50 °C for 28 h before use. The DSC studies show that the antimicrobial PUII films exhibit good thermal properties. The antimicrobial PUII films show two Tgs; the lower temperature shows the soft segment Tg (−20.9 °C) and the higher temperature shows the hard segment Tg (10 °C).

### 3.5. Antimicrobial Properties

**Figure 7:**
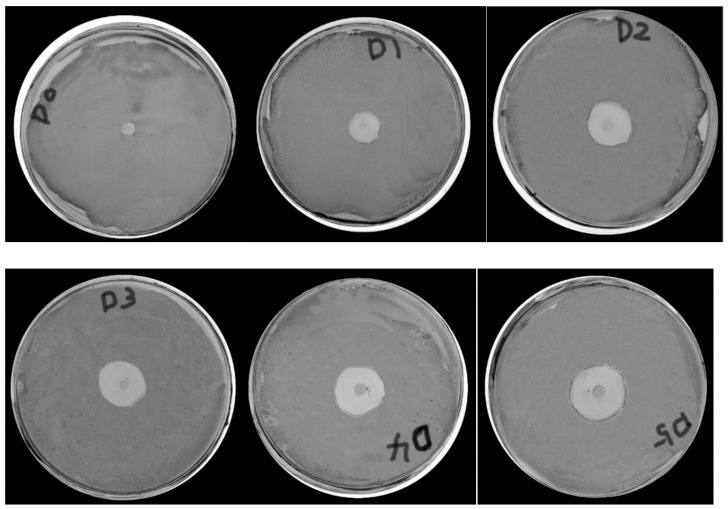
The inhibiting capacity of films with different amounts (0%, 0.2%, 0.4%, 0.6%, 0.8%, 1.0%) of 1,3,5-Tris(2-hydroxyethyl)hexahydro-1,3,5-triazine (TNO) on *Escherichia coli* (D_0_: TNO% = 0%; D_1_: TNO%=0.2%; D_2_: TNO% = 0.4%; D_3_: TNO% = 0.6%; D_4_: TNO% = 0.8%; D_5_: TNO% = 1.0%).

**Figure 8:**
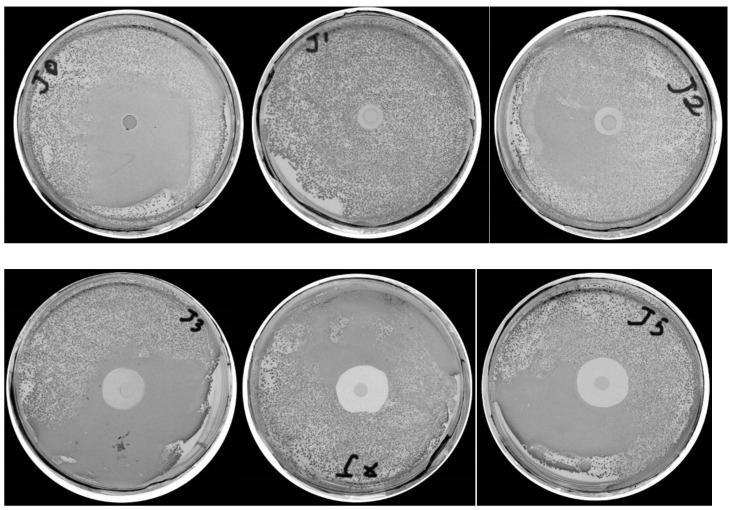
The inhibiting capacity of films with different amounts (0%, 0.2%, 0.4%, 0.6%, 0.8%, 1.0%) of 1,3,5-Tris(2-hydroxyethyl)hexahydro-1,3,5-triazine (TNO) on *Staphylococcus aureus* (J_0_: TNO% = 0%; J_1_: TNO% = 0.2%; J_2_: TNO% = 0.4%; J_3_: TNO% = 0.6%; J_4_: TNO% = 0.8%; J_5_: TNO% = 1.0%).

**Figure 9:**
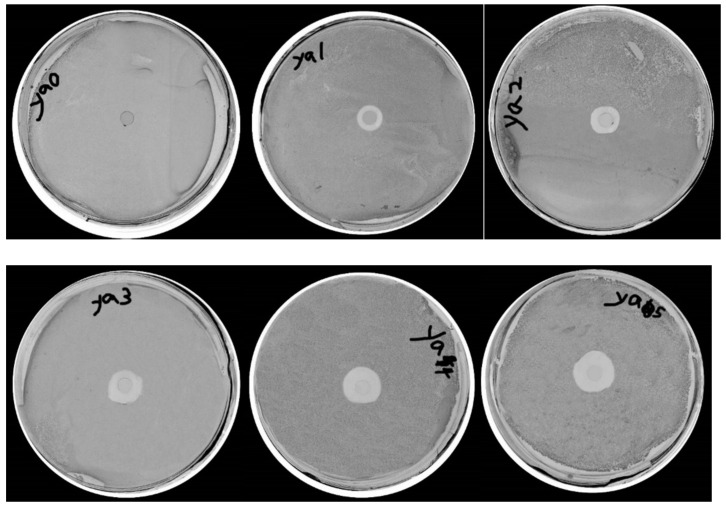
The inhibiting capacity of films with different amounts (0%, 0.2%, 0.4%, 0.6%, 0.8%, 1.0%) of 1,3,5-Tris(2-hydroxyethyl)hexahydro-1,3,5-triazine (TNO) on *Bacillus subtilis* (ya_0_: TNO% = 0%; ya_1_: TNO% = 0.2%; ya_2_: TNO% = 0.4%; ya_3_: TNO% = 0.6%; ya_4_: TNO% = 0.8%; ya_5_ TNO% = 1.0%).

**Figure 10:**
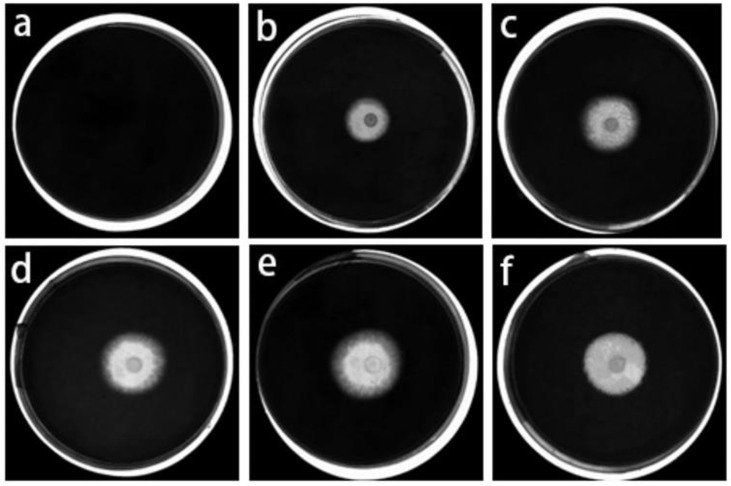
The inhibiting capacity of films with different amounts (0%, 0.2%, 0.4%, 0.6%, 0.8%, 1.0%) of 1,3,5-Tris(2-hydroxyethyl)hexahydro-1,3,5-triazine (TNO) on *Gray mold* ((**a**): TNO% = 0%; (**b**): TNO% = 0.2%; (**c**): TNO% = 0.4%; (**d**): TNO% = 0.6%; (**e**): TNO% = 0.8%; (**f**): TNO% = 1.0%).

The inhibition zone method was employed. The PUII films with different amounts (0.2%, 0.4%, 0.6%, 0.8%, 1.0%) of 1,3,5-Tris(2-hydroxyethyl)hexahydro-1,3,5-triazine (TNO) were challenged with *Escherichia coli*, *Staphylococcus aureus*, *Bacillus subtilis* and *Gray mold*, as shown in Table 3 and Figure 7, Figure 8, Figure 9 and Figure 10. The results show that when a small amount of antibacterial agent was added, the antibacterial effect of films on Botrytis cinerea was more obvious. When more antibacterial agent was added, the films showed a better inhibitory effect on *Staphylococcus aureus* and *Escherichia coli*. In addition, the inhibitory effect on the *Bacillus subtilis* was not effective.

## 4. Conclusions

Novel physically crosslinked polyurethane (PUII) based on isophorone diisocyanates was prepared by a conventional two-step method. The chemical structures of the PUII were characterized by FTIR, ^1^H NMR, GPC, SEM and DSC. The self-assembly behavior of PUII has also been tested, and the results showed that the micron materials created irregular globular morphologies. This investigation provides a clear insight into the solvent-induced self-assembly in the novel PUII polymer hydrogels for the various morphologies and sizes ranging from micron- to nanometer-sized pores and spheres. The antimicrobial property of the PUII films containing an antibacterial agent was examined against *Escherichia coli*, *Staphylococcus aureus*, *Bacillus subtilis* and *Gray mold* and showed good antibacterial properties. The mechanical evaluation shows that polyurethane (PUII) materials exhibit good mechanical properties. The new PUII has good mechanical properties, good antibacterial properties, and poor barrier properties compared with other polyurethanes.

## Figures and Tables

**Table 1:** The transmittance and haze of the PUII films.

The Content of Antibacterial Agent (%)	Transmittance (%)	Haze (%)
0	89.7	51.11
0.2	73.5	90.23
0.4	70.2	90.31
0.6	66.3	91.22
0.8	62.8	92.13
1.0	61.1	92.91

**Table 2:** Mechanical performance of the PUII films.

The Content of Antibacterial Agent (%)	Breaking Elongation (%)	Tensile Strength (MPa)
0 ave. ^a^	502.7 ± 40.1	41.1 ± 3.2
0.2 ave.	519.9 ± 43.2	42.3 ± 3.5
0.4 ave.	528.8 ± 47.5	43.5 ± 4.2
0.6 ave.	539.6 ± 45.3	44.2 ± 3.8
0.8 ave.	557.8 ± 48.6	45.1 ± 5.2
1.0 ave.	581.9 ± 52.8	46.9 ± 4.7

^a^ The average number of 5 films. Data are expressed as mean ± SD.

**Table 3:** The antibacterial circle diameter of different samples.

The Content of Antibacterial Agent/%	The Antibacterial Circle Diameter/mm			
	*Escherichia coli*	*Staphylococcus aureus*	*Bacillus subtilis*	*Gray mold*
0	0	0	0	0
0.2	14.1	10.2	10.3	17.8
0.4	18.1	11.7	11.9	18.5
0.6	19.3	18.3	13.8	19.1
0.8	20.9	21.9	15.5	19.9
1.0	23.2	22.1	17.1	22.2

## Data Availability

The authors declare data availability.
